
JOURNAL INFORMATION
==============================
NLM Title Abbreviation: Medizinrecht
Journal ID: Medizinrecht
NLM Journal ID: 8309354

Medizinrecht

ISSN: 0723-8886
EISSN: 1433-8629

ARTICLE INFORMATION
==============================
PMCID: PMC9778459
PMID: 36575718
DOI: 10.1007/s00350-022-6355-y
Article ID: 6355
Article version: 1
Subjects: Aufsätze

Rechtsprechungsübersicht zum Medizinprodukterecht und zu angrenzenden Gebieten 2021/2022

Hobusch Sandra 1
Ochs Sebastian 2
1 grid.461772.1 0000 0004 0374 5032 Ostfalia Hochschule für angewandte Wissenschaften, Rothenfelder Straße 10, 38440 Wolfsburg, Deutschland
2 Grünecker Patent- & Rechtsanwälte, Leopoldstraße 4, 80802 München, Deutschland
Electronic publication date: 2022 Dec 22
Print publication date: 2022
Volume: 40
Issue: 12
First page: 992
Last page: 999
Copyright: © Der/die Autor(en) 2022
License: Open Access Dieser Artikel wird unter der Creative Commons Namensnennung 4.0 International Lizenz veröffentlicht, welche die Nutzung, Vervielfältigung, Bearbeitung, Verbreitung und Wiedergabe in jeglichem Medium und Format erlaubt, sofern Sie den/die ursprünglichen Autor(en) und die Quelle ordnungsgemäß nennen, einen Link zur Creative Commons Lizenz beifügen und angeben, ob Änderungen vorgenommen wurden.
Die in diesem Artikel enthaltenen Bilder und sonstiges Drittmaterial unterliegen ebenfalls der genannten Creative Commons Lizenz, sofern sich aus der Abbildungslegende nichts anderes ergibt. Sofern das betreffende Material nicht unter der genannten Creative Commons Lizenz steht und die betreffende Handlung nicht nach gesetzlichen Vorschriften erlaubt ist, ist für die oben aufgeführten Weiterverwendungen des Materials die Einwilligung des jeweiligen Rechteinhabers einzuholen.
Weitere Details zur Lizenz entnehmen Sie bitte der Lizenzinformation auf http://creativecommons.org/licenses/by/4.0/deed.de.
License URL: https://creativecommons.org/licenses/by/4.0/

issue-copyright-statement: © C.H. Beck Verlag oHG, München und Springer-Verlag GmbH Deutschland, ein Teil von Springer Nature 2022

==============================

**Sandra Hobusch und Sebastian Ochs**

## 1. Vorbemerkungen

Die nachfolgende Rechtsprechungsübersicht knüpft an den Vorjahresbericht von *Hobusch/Ochs*, MedR 2021, 886 ff. an. Sie gibt erneut einen Überblick über Medizinprodukte in der Rechtsprechung zum Medizinprodukte-, Wettbewerbs-, Haftungs- und Krankenversicherungsrecht.

## 2. Medizinprodukterecht

### *a) Begriff und Einstufung*

Das BVerwG[1] setzte zwei Verfahren aus und legte dem EuGH jeweils verschiedene Fragen zur Vorabentscheidung vor. Ein Fragenkomplex betrifft die pharmakologische Wirkung als Abgrenzungsmerkmal zwischen Arzneimitteln und Medizinprodukten. Das Bundesinstitut für Arzneimittel und Medizinprodukte (BfArM) hatte ein als stoffliches Medizinprodukt in den Verkehr gebrachtes Nasenspray als zulassungspflichtiges Arzneimittel eingestuft. Das Nasenspray ist zur Reinigung und Drainage der mit Schleim und Sekret gefüllten Nasenhöhlen bestimmt und führt zu einem intensiven Sekretabfluss und der damit einhergehenden Symptomerleichterung bei Stauungszuständen in der Nase. Ein anderer Bescheid des BfArM betraf Nasentropfen, die ebenfalls als stoffliches Medizinprodukt in den Verkehr gebracht worden waren. Die Nasentropfen wurden als Mittel zur unterstützenden Behandlung bei Schnupfen und zur begleitenden Behandlung bei Reizungen der Nasenschleimhaut mit Sekretbildung beworben. Diese Nasentropfen stufte das BfArM ebenfalls mit Hinweis auf die pharmakologische Wirkung als Arzneimittel ein. Bei der pharmakologischen Wirkung geht es um eine Wechselwirkung zwischen den Molekülen der in Frage stehenden Substanz und einem zellulären Bestandteil (Rezeptor), wobei es nach der Rechtsprechung des EuGH[2] genügt, dass die Wechselwirkung zwischen der Substanz und einem beliebigen sich im Körper des Anwenders befindlichen zellulären Bestandteils stattfindet. Damit werden nicht nur die Wechselwirkungen mit den zellulären Bestandteilen des Menschen, sondern auch die mit den im menschlichen Körper befindlichen Bakterien oder Viren erfasst, weil auf diesem Weg die physiologischen Funktionen des Menschen ebenfalls wiederhergestellt, korrigiert oder beeinflusst werden können. In derartigen Fällen sind nach Ansicht des BVerwG die Kriterien für die Abgrenzung der pharmakologischen und nicht-pharmakologischen Mittel nicht hinreichend geklärt. Deshalb hat das BVerwG dem EuGH die Frage vorgelegt, ob die bestimmungsgemäße Hauptwirkung eines Stoffes auch dann pharmakologisch sein kann, wenn sie nicht auf einer rezeptorvermittelten Wirkweise beruht und die Substanz vom menschlichen Körper auch nicht absorbiert wird, sondern an der Oberfläche etwa von Schleimhäuten verbleibt und dort reagiert. Ferner möchte das BVerwG vom EuGH wissen, ob ein Erzeugnis als stoffliches Medizinprodukt angesehen werden kann, wenn die Wirkweise des Erzeugnisses nach dem Stand der Wissenschaft offen ist und deshalb nicht abschließend geklärt ist, ob die bestimmungsgemäße Hauptwirkung auf phar-

Prof. Dr. iur. Sandra Hobusch,
Ostfalia Hochschule für angewandte Wissenschaften,
Rothenfelder Straße 10, 38440 Wolfsburg, Deutschland

Rechtsanwalt Sebastian Ochs,
Grünecker Patent- & Rechtsanwälte,
Leopoldstraße 4, 80802 München, Deutschland

1) BVerwG, Beschl. v. 20. 5. 2021 – 3 C 9.20 –, PharmR 2021, 593 ff., anhängig EuGH C-496/21; BVerwG, Beschl. v. 20. 5. 2021 – 3 C 19/19 –, juris, anhängig EuGH C-495/21.
2) EuGH, Urt. v. 6. 9. 2012 – C-301/11 –, PharmR 2012, 442 ff.

makologischem oder physikalisch-chemischem Weg erzielt wird. Des Weiteren hat das BVerwG dem EuGH die Frage zur Vorabentscheidung vorgelegt, ob ein stoffliches Medizinprodukt auch ein Präsentationsarzneimittel gem. Art. 1 Nr. 2 Buchst. a RL 2001/83/EG (nationale Umsetzung: § 2 Abs. 1 S. 2 Nr. 1 AMG) sein kann. Hintergrund für diese Frage ist, dass ein Medizinprodukt ebenso wie ein Präsentationsarzneimittel eine therapeutische Zweckbestimmung hat und es nicht auszuschließen ist, dass die dem Produkt beigefügte Gebrauchsanweisung und die Produktpackung beim maßgeblichen durchschnittlich informierten, aufmerksamen und verständigen Verbraucher den Eindruck eines Arzneimittels erwecken.

Das OLG Frankfurt a. M.[3] berief sich in einem wettbewerbsrechtlichen Verfahren bei der Einstufung eines Hustensaftes (konkret: Mucosolvan Phyto Complete) als Medizinprodukt auf die Tatbestandwirkung des Bescheides des BfArM. Das BfArM hatte auf Antrag einer Landesbehörde gem. § 21 Abs. 4 AMG beschieden, dass der in Rede stehende Hustensaft kein zulassungspflichtiges Arzneimittel sei. Das OLG führte aus, dass § 21 Abs. 4 AMG dem BfArM die Befugnis verleihe, den Produktstatus aus Gründen der Rechtssicherheit festzulegen, und die Rechtsauffassung des BfArM vertretbar sei. Die bindende Tatbestandswirkung würde erst entfallen, wenn der Bescheid, der einen Verwaltungsakt darstellt, nichtig sei. Dafür sah das OLG keinen Anhaltspunkt.

Das VG Köln[4] wies die Klage gegen einen Bescheid des BfArM, mit dem Heiltorf als Medizinprodukt eingestuft worden war, als unbegründet ab. Es verwies auf die vor allem thermophysikalischen Eigenschaften des Torfes respektive des Moors der Klägerin. In einem anderen, ein Solewasser betreffendes Verfahren schloss sich das VG Köln ebenfalls der Auffassung des BfArM zur Einstufung des Heilwassers als Medizinprodukt an[5]. Das VG führte aus, dass die bestimmungsgemäße Hauptwirkung des Heilwassers sowohl bei der Anwendung als Solebad als auch zur Inhalation physikalisch erreicht werde. Die Anwendung als Solebad zur Behandlung von degenerativen und funktionellen Wirbelsäulen- und Gelenkerkrankungen und entzündlich-rheumatischen Erkrankungen knüpft an den stärkeren Auftrieb des Körpers in einem Natriumchlorid haltigen Wasser und der damit einhergehenden Entlastung der Gelenke und Muskeln an. Soweit die Solebäder bei Hauterkrankungen zur Anwendung kommen, werde das im Wasser enthaltene Natriumchlorid auf der Hautfläche abgelagert und rufe einen Reizzustand der Haut hervor, auf den der Körper kurzfristig mit einer Gegenregulation und langfristig mit einer Adaption reagiere. Diese physiologischen Gegenreaktionen auf physikalische Reize würden jedoch keine pharmakologische Wirkung des Solewassers belegen. Hinsichtlich der Anwendung des Heilmittels zur Inhalation ergab sich für das VG aus dem vorgelegten Gutachten ebenfalls kein nachgewiesener arzneilicher Wirkmechanismus. Das VG schloss sich der Auffassung des BfArM an, dass die positiven klinischen Effekte auf physikalische Vorgänge, insbesondere der Osmose, zurückzuführen seien. Die Inhalation des Natriumchlorid haltigen Wassers führe zu einer Verflüssigung des Bronchialschleims und damit verbunden zu einem verbesserten Abtransport[6].

### b) Zweckbestimmung

Wenn die Gebrauchsanleitung einer Krankentrage vorsieht, dass diese nur für den Transport von Erkrankten und Verletzten innerhalb und außerhalb von Rettungswagen und Krankentransportwagen einzusetzen ist, so handelt es sich dabei um eine Zweckbestimmung. Wenn die Krankentrage nicht in den genannten hinsichtlich ihrer Eigenschaften und Ausstattung den EN- und DIN-Normen unterliegenden Rettungsmitteln, sondern in einem lediglich dem § 49 PBefG unterliegenden Mietliegewagen eingesetzt wird, widerspricht es ihrer Zweckbestimmung. Das hat zur Folge, dass gegen § 4 Abs. 1 MPBetreibV verstoßen wird, weil danach Medizinprodukte nur ihrer Zweckbestimmung entsprechend und nach den Vorschriften dieser Verordnung sowie den allgemein anerkannten Regeln der Technik betrieben und angewendet werden dürfen. Da § 4 Abs. 1 MPBetreibV zudem eine das Marktverhalten regelnde Vorschrift i. S. des § 3a UWG ist, verurteilte das OLG Hamm[7] einen Mietwagenunternehmer im Rahmen eines wettbewerbsrechtlichen Verfahren, es zu unterlassen, die Krankentrage in seinen Fahrzeugen einzusetzen.

### c) Inverkehrbringen und Abgabe von Medizinprodukten

Das OVG Schleswig-Holstein[8] bestätigte in einem Verfahren des einstweiligen Rechtsschutzes (wie die Vorinstanz[9]) die Rechtmäßigkeit einer behördlichen Verfügung v. 3. 12. 2020, mit der einer Herstellerin von medizinischen Einmal-Sets für die Diagnostik und Behandlung kardiologischer und ophthalmologischer Erkrankungen das Inverkehrbringen von bestimmten steril verpackten Einmal-Sets mit einer CE-Kennzeichnung untersagt worden war, weil sie für den Sterilisationsprozess kein gültiges Zertifikat habe. Die Herstellerin berief sich auf das Zertifikat einer Benannten Stelle v. 27. 8. 2018 mit Gültigkeit bis zum 26. 8. 2023 und auf die Übergangsvorschrift in Art. 120 VO (EU) 2017/745 über Medizinprodukte (MDR), nach der die nach der RL 93/42/EWG zertifizierten Produkte unter bestimmten Voraussetzungen in einer Übergangsphase weiterhin in den Verkehr gebracht werden dürfen. Nach Art. 120 Abs. 1 MDR ist eine nach der RL 93/42/EWG erteilte Benennung einer Benannten Stelle ab 26. 5. 2021 ungültig. Konformitätsbescheinigungen, die eine solche Benannte Stelle nach dem 25. 5. 2017 ausgestellt hat, bleiben jedoch gem. Art. 120 Abs. 2 S. 2 MDR bis zum Ende des in der Bescheinigung genannten Zeitraums, max. fünf Jahre ab Ausstellung, gültig, damit die Produkte in der Übergangsphase von der RL 93/42/EWG zur MDR in den Verkehr gebracht werden können. Ferner bestimmt Art. 120 Abs. 3 MDR, dass ein Medizinprodukt mit einer nach Abs. 2 weitergeltenden Bescheinigung bis zum 26. 5. 2024 auch dann in Verkehr gebracht werden kann, wenn das Produkt nicht den Anforderungen der MDR entspricht, sofern es ab dem 26. 5. 2021 weiterhin der RL 93/42/EWG entspricht und sofern keine wesentlichen Änderungen der Auslegung und der Zweckbestimmung vorliegen. Im Hinblick auf diesen Abs. 3 stellte das OVG Schleswig-Holstein klar, dass er nur zur Anwendung kommt, wenn die Benannte Stelle ihre Notifizierung nach Art. 120 Abs. 1 MDR verloren habe. Das ergebe sich zum einen aus der Bezugnahme von Abs. 3 auf Abs. 2 und von Abs. 2 auf Abs. 1. Zum anderen solle Art. 120 MDR nicht ermöglichen, dass Produkte, die bei Fortgeltung der RL 93/42/EWG nicht verkehrsfähig gewesen wären, durch das Inkrafttreten der MDR in den Verkehr gebracht werden dürfen. Im Fall der Herstellerin der steril verpackten Einmal-Sets hatte die für das Konfor-

3) OLG Frankfurt a. M., Urt. v. 8. 7. 2021 – 6 U 126/20 –, PharmR 2021, 560 ff.; anders OLG Frankfurt a. M., Urt. v. 22. 5. 2020 – 6 U 23/20 –, GRUR-RR 2020, 456 ff., als das beklagte Pharmaunternehmen keinen ungeschwärzten Bescheid vorlegte, vgl. Vorjahresbericht *Hobusch/Ochs*, MedR 2021, 886 ff., sub. 2.a.
4) VG Köln, Urt. v. 18. 5. 2021 – 7 K 6491/18 –, juris.
5) VG Köln, Urt. v. 18. 5. 2021 – 7 K 6492/18 –, juris.
6) Ebd., Rdnr. 36.
7) OLG Hamm, Urt. v. 24. 6. 2021 – 4 U 184/20 –, NJW-RR 2021, 1341 ff.
8) OVG Schl.-Holst., Beschl. v. 23. 9. 2021 – 3 MB 22/21 –, PharmR 2022, 23 ff.
9) Schl.-Holst. VG, Beschl. v. 23. 6. 2021 – 1 B 167/20 –, juris.

mitätsverfahren eingeschaltete Benannte Stelle ihre Notifizierung jedoch nicht nach Art. 120 Abs. 1 MDR verloren. Die Benennung war bereits vorher aufgrund einer mangelhaften Aufgabenwahrnehmung erloschen, so dass die Herstellerin nicht über ein gültiges Zertifikat verfügte und die behördliche Untersagungsverfügung zurecht erfolgte.

Gem. § 3 Abs. 1 S. 2 MPAV dürfen Medizinprodukte, die nicht zur Anwendung durch Laien vorgesehen sind, nur an Fachkreise abgegeben werden, es sei denn, dass eine ärztliche oder zahnärztliche Verschreibung vorgelegt wird. Zu den Fachkreisen gehören Angehörige der Heilberufe oder des Heilgewerbes oder Angehörige von Einrichtungen, die der Gesundheit dienen, sowie sonstige Personen, die Medizinprodukte herstellen, prüfen, in der Ausübung ihres Berufs in den Verkehr bringen, implantieren, in Betrieb nehmen, betreiben oder anwenden (§ 3 Nr. 2 MPDG). Wenn „Geschäftskunden" nicht zur Anwendung durch Laien bestimmte Medizinprodukte (im Konkreten: Dermaroller mit Nadellängen von 2–3 mm für die sog. Needlingtherapie) über einen Online-Shop beziehen können, genügt es für die Überprüfung der Zugehörigkeit zu den Fachkreisen nicht, wenn bei der Onlineregistrierung und -anmeldung nur die Umsatzsteuer-Identifikationsnummer angegeben werden muss. Die Besteller können allen Branchen angehören und die Produkte unter Nutzung ihrer beruflichen Umsatzsteuer-Identifikationsnummer für den privaten Gebrauch erwerben. Ein derartiger Vertrieb ist unzulässig, so dass eine diesbezügliche behördliche Untersagungsverfügung vom VG Berlin[10] als rechtmäßig angesehen wurde. Ferner bestätigte das VG die Rechtmäßigkeit des behördlichen Vertriebsverbots hinsichtlich der Dermaroller mit einer Nadellänge von 0,3 bis 1,5 mm, die zur Anwendung durch Laien vorgesehen waren. Für diese Produkte sahen die Gebrauchshinweise vor, dass sie vor und nach jeder Behandlung gereinigt, desinfiziert und sterilisiert werden sollen. Diese Aufbereitung ist notwendig, um das Infektionsrisiko auszuschließen. Die Nadeln der Dermaroller durchstechen die obere(n) Hautschicht(en), um über die Einstiche Wirkstoffe in die Haut einzubringen. Insoweit führte das VG aus, dass nicht davon auszugehen sei, dass einem durchschnittlichen Verbraucher insbesondere die Sterilisation möglich ist. Selbst wenn der Verbraucher die dafür notwendigen Geräte anschaffen würde, sei eine ordnungsgemäße Sterilisation keinesfalls sichergestellt. Damit würden die Produkte nicht den unionsrechtlichen Sicherheits- und Leistungsanforderungen erfüllen. Insbesondere läge ein Verstoß gegen Kapitel II Nr. 11.2 des Anhangs I der Verordnung (EU) 2017/745 vor, wonach das Produkt erforderlichenfalls so auszulegen ist, dass es leicht zu reinigen, zu desinfizieren und/oder zu sterilisieren ist. Wegen der Nichtkonformität der Produkte und der damit einhergehenden Risiken für die Sicherheit und Gesundheit der Verbraucher habe die Behörde zurecht den Vertrieb der Produkte untersagt.

*d) Medizinproduktrechtliche Anforderungen an SARS-CoV-2-Teststellen*

Bei den Coronavirustests nach § 1 TestV[11] handelt es sich um In-vitro-Diagnostika, so dass das Medizinproduktegesetz (MPG) in der bis 25. 5. 2021 geltenden Fassung bis zum 25. 5. 2022[12] sowie die Medizinprodukte-Betreiberverordnung (MPBetreibV) anzuwenden sind. Gem. § 9 Abs. 1 MPBetreibV hat derjenige, der laboratoriumsmedizinische Untersuchungen durchführt, vor Aufnahme dieser Tätigkeit ein Qualitätssicherungssystem nach dem Stand der medizinischen Wissenschaft und Technik zur Aufrechterhaltung der erforderlichen Qualität, Sicherheit und Leistung bei der Anwendung von In-vitro-Diagnostika sowie zur Sicherstellung der Zuverlässigkeit der damit erzielten Ergebnisse einzurichten. Eine ordnungsgemäße Qualitätssicherung wird vermutet, wenn Teil A der Richtlinie der Bundesärztekammer zur Qualitätssicherung laboratoriumsmedizinischer Untersuchungen (Rili-BÄK)[13] beachtet wird. Diese Richtlinie verlangt u. a. eine eindeutige Festlegung und nachvollziehbare Dokumentation der Verantwortung und Zuständigkeit für die Durchführung der Aufgaben (Ziff. 4.2 Rili-BÄK) sowie geeignete Räume, die weder die Durchführung der laboratoriumsmedizinischer Untersuchungen beeinträchtigen noch die Gesundheit und Sicherheit der Patienten und Mitarbeiter gefährden (Ziff. 5.3.1 Rili-BÄK). Das Hygienekonzept sowie die unordentlichen, verschmutzten und schwer zu reinigenden Räumlichkeiten einer Zahnärztin erfüllten, wie eine Begehung der Teststelle sowie eine wenige Tage später erfolgte Inspektion durch das Gesundheitsamt ergab, nicht die genannten Anforderungen, so dass das zuständige Landesamt der Zahnärztin auf der Grundlage des § 28 Abs. 1, 2 S. 1, 2 MPG die Durchführung der Coronavirustests untersagte und die sofortige Vollziehung der Untersagungsverfügung anordnete. Den Antrag der Zahnärztin auf Wiederherstellung der aufschiebenden Wirkung des Widerspruchs lehnte das VG Neustadt (Weinstraße)[14] ab, weil es den behördlichen Bescheid für offensichtlich rechtmäßig hielt.

*e) Risikoabwehr*

Nach dem mittlerweile außer Kraft getretenen § 21 Abs. 3 MPSV durften die zuständigen Bundesoberbehörden Informationen und Auskünfte zu vorliegenden Meldungen von Vorkommnissen und durchgeführten Rückrufen sowie zu durchgeführten Risikobewertungen und korrektiven Maßnahmen an den Medizinischen Dienst des Spitzenverbandes Bund der Krankenkassen, die Deutsche Krankenhausgesellschaft und andere Organisationen, Stellen und Personen übermitteln, soweit von diesen ein Beitrag zur Risikoverringerung geleistet werden konnte oder ein berechtigtes Interesse bestand. Auf dieser Grundlage verlangten Krankenkassen vom BfArM die Herausgabe einer Kundenliste eines Unternehmens, das Hüftendoprothesen vertrieb. Das BfArM hatte die Hüftendoprothesen einer Risikobewertung unterzogen, die zum Anwendungsstopp bzw. zur Entscheidung des Unternehmens, die Produkte aus dem Vertrieb zu nehmen, führte. Die Krankenkassen begehrten die Kundenliste, die auch die belieferten Kliniken in Deutschland enthielt, um Schadenersatzansprüche prüfen und geltend machen zu können. Das BfArM lehnte die Herausgabe der Kundenliste ab. Die dagegen gerichtete Klage wies das VG Köln ab. Das OVG Nordrhein-Westfalen[15] verurteilte das BfArM zur Neubescheidung der klagenden Krankenkassen. Nun stellte das BVerwG[16] das erstinstanzliche Urteil wieder her. Es führte aus, dass bei der Entscheidung über ein Auskunftsbegehren auf die zum Zeitpunkt der gerichtlichen Entscheidung geltende Rechtslage abzustellen sei, so dass der außer Kraft getretene § 21 Abs. 3 MPSV nicht mehr anwendbar sei. Eine andere

10) VG Berlin, Urt. v. 10. 1. 2022 – 14 K 792.17 –, PharmR 2022, 404 ff.

11) Verordnung zum Anspruch auf Testung in Bezug auf einen direkten Erregernachweis des Coronavirus SARS-CoV-2 v. 21. 9. 2021, BAnz. AT 21. 9. 2021 V1.

12) Vgl. § 2 Abs. 1 Gesetz zur Durchführung unionsrechtlicher Vorschriften betreffend Medizinprodukte (MPDG) v. 28. 4. 2020, BGBl. I S. 960.

13) DÄBl., Jg. 111, Heft 38 v. 19. 9. 2014, S. A 1583; Neufassung: DÄBl., Jg. 116, Heft 51–52 v. 23. 12. 2019, S. A 2422.

14) VG Neustadt (Weinstraße), Beschl. v. 7. 1. 2022 – 5 L 1239/21.NW –, juris.

15) OVG Nordrh.-Westf., Urt. v. 19. 11. 2019 – 13 A 1326/17 –, MPR 2020, 52 ff.; vgl. auch *Hobusch/Ochs*, MedR 2020, 911 ff., sub. 2.b.

16) BVerwG, Urt. v. 14. 7. 2021 – 3 C 2/20 –, NVwZ 2021, 1866 ff.

(Nachfolge-)Vorschrift, auf die der Auskunftsanspruch gestützt werden könne, existiere nicht. Im Übrigen handele es sich bei der Kundenliste nicht um eine Information i. S. des § 22 Abs. 3 MPSV. Die Vorschrift habe nur Informationen zum Ergebnis der Risikobewertung erfasst. Die Risikobewertung habe sich jedoch im konkreten Fall nicht auf die Kundenliste erstreckt, weil sie bei der Risikobewertung zunächst nicht vorgelegen habe, und nachdem sie unaufgefordert von der Landesbehörde, die das Vertriebsunternehmen überwachte, übermittelt worden war, nicht einbezogen worden sei.

### 3. Medizinprodukte und Wettbewerbsrecht

*a) Irreführung*

Unter Rückgriff auf die besonders strengen Anforderungen an die Richtigkeit, Eindeutigkeit und Klarheit von Werbeaussagen bei gesundheitsbezogener Werbung entschied das KG[17], dass eine Werbeaussage irreführend ist, wenn diese sich auf Studien stützt, die diese Aussage nicht tragen. Insoweit, so der Senat, komme es auch nicht darauf an, dass ggfs. andere Studien die Aussage als inhaltlich richtig belegen können. Denn der angesprochene Verkehr, hier Ärzte, werde in seinem Vertrauen enttäuscht, die Aussage durch die in Bezug genommene Studie überprüfen zu können. Auf diese Weise komme es zu einer Beeinträchtigung der Sicherheit ärztlicher Therapieentscheidung. Es liege daher in diesem Fall grundsätzlich eine relevante Irreführung vor. Die Antragsgegnerin bewarb Hyaluron-Natrium-Fertigspritzen im Rahmen eines Programmhefts für Ärzte mit der Behauptung „Hemmt effektiv die Entzündung". Sie bezog sich ausdrücklich auf eine Studie, die sich jedoch zu dieser Wirkung gar nicht verhielt. Genauso lag der Fall in Bezug auf die Anpreisung, dass das Produkt „die körpereigene Hyaluronsäure-Synthese anrege". Das KG sah darin einen Verstoß gegen § 3 S. 1 S. 2 HWG.

In einem Hinweisbeschluss äußerte das OLG Düsseldorf[18] sich zu dem Verständnis der angesprochenen Verkehrskreise von dem Begriff „hypoallergen" in Bezug auf Produkte zur Wundversorgung. Die in Rede stehende Werbung richtete sich dabei sowohl an Fachleute als auch an Verbraucher. Der Senat kam zu dem Ergebnis, dass der angesprochene Verkehr den Begriff „hypoallergen" nicht in dem engen Sinn „nicht allergen" auf Basis seiner wörtlichen Übersetzung von „unterhalb der Allergie auslösenden Schwelle" versteht. Das relevante allgemeine Verständnis dieses Begriffs geht dahin, dass ein Produkt bloß weniger allergene Stoffe beinhalte. Dies stütze die Internetrecherche, so der Senat. Dort werden eine Vielzahl von Produkten mit dem Begriff „hypoallergen" angepriesen. Diese weisen aber erkennbar nur weniger allergene Stoffe auf, nicht aber gar keine. Auch die Recherche nach dem Begriff „hypoallergen" ergebe, dass darunter solche Substanzen verstanden werden, die weniger allergische Reaktionen hervorrufen. Zwischen den Parteien war nämlich unstreitig, dass das in Rede stehende beworbene Produkt weniger allergene Substanzen aufweise, als herkömmliche Produkte zur Wundversorgung. Demzufolge konnte das Gericht auch keine Irreführung erkennen.

Das OLG Hamm[19] sah die Bewerbung von Mineralien mit den Begriffen „Heilstein" oder „EFM-Strahlenschutz" als irreführend gem. § 3, 3a UWG, § 3 S. 1, S. 2 HWG an. Den Produkten, die mit einem Mineral versehen waren, werde damit eine prophylaktische sowie therapeutische Wirksamkeit gegenüber elektromagnetischer Strahlung zugesprochen. Diese weise das Produkt jedoch nicht auf. Insbesondere fehlten, so der Senat, jegliche wissenschaftlichen Erkenntnisse für diese behauptete Wirkung.

Für die Einstufung einer Werbung von Medizinprodukten als irreführend gelte das europäische Irreführungsverbot für Medizinprodukte gem. Art. 7 MDR, so das OLG Frankfurt a. M.[20]. Es hielt fest, dass die Übergangsregel gem. Art. 120 Abs. 4 MDR mit dem darin enthaltenen Tatbestandsmerkmal der rechtmäßigen Inverkehrbringung auf die Zertifizierung abstelle, nicht hingegen irreführende Werbeangaben für Produkte legitimieren soll, die vor dem Stichtag 26. 5. 2020 über eine Zertifizierung verfügten. Weiter hielt der Senat fest, dass die Kriterien, die zu § 3 HWG entwickelt wurden, auch auf Art. 7a MDR angewendet werden können. Es gelten insoweit die gleichen Anforderungen an Wirkungsaussagen, so der Senat. In diesem Sinne entschied das OLG, dass die Bewerbung von Heilerde mit u. a. der Unterstützung bei der Körperentgiftung, -entschlackung und -reinigung, der Bindung freier Radikale und Unterstützung der Histamin-Intoleranz nach dem sog. Strengeprinzip irreführend sei und nicht der Wahrheit entspreche. Die Angaben seien, so der Senat, wissenschaftlich ungesichert bzw. jedenfalls fachlich umstritten. Mangels Bildung von Schlacken könne von keiner Entschlackung eines Körpers gesprochen werden. Die entgiftende Wirkung sei jedenfalls fachlich umstritten und hinsichtlich der Histamin-Intoleranz fehle jegliche wissenschaftliche Absicherung. Die Beklagte konnte keine Nachweise für die wissenschaftliche Grundlage in Bezug auf die Bindung freier Radikale liefern und auch sonst keine Studienergebnisse hierzu vorlegen. Ein weniger strenger Maßstab sei auch nicht deshalb anzusetzen, weil das in Rede stehende Medizinprodukt vom Körper nicht resorbiert werde. Bloße physikalische Wirkungen änderten nichts daran, dass zum Schutz der Verbraucher das Strengeprinzip zur Anwendung komme. Im Hinblick auf das Angebot von Zeugenbeweis anstelle von Studienergebnissen hielt der Senat fest, dass derartigem Beweisangebot nicht nachzugehen sei, da dieser keine Studien ersetzen könne. Art. 7 MDR stelle eine Marktverhaltensregel gemäß § 3a UWG dar, weshalb das OLG die Werbung als unlauter einstufte. Das OLG Hamm[21] schloss sich der Rechtsprechung des OLG Frankfurt a. M. an und sah in Art. 7 MDR ebenfalls eine Marktverhaltensregel gem. § 3a UWG. Wie das OLG Frankfurt a. M. wendet auch das OLG Hamm die Rechtsprechung zu § 3 S. 2 Nr. 2 HWG auf Art. 7 MDR 5 an. In dem vom OLG Hamm zu entscheidenden Fall ging es um Produkte zur Wundversorgung. Auch nach dem OLG Hamm ist das Strengeprinzip auf Medizinprodukte, die nur physikalisch wirken und nicht vom Körper resorbiert werden anzuwenden. Wird diesen heilende Wirkung beigemessen, so muss diese Behauptung entsprechend abgesichert sein. Der angesprochene Verkehr versteht die Angabe „Anwendungsbereich: Infizierte Wunden – Wundstarrinfektion" dahingehend, dass die Wundauflage für die Körpergesundheit eine bestimmte Wirkung habe, nämlich in Bezug auf den Heilungsprozess infizierter Wunden. Nachdem neben Fachleuten auch bestimmte Verbraucher zu dem angesprochenen Verkehrskreis gehörten, reiche es aus, wenn jedenfalls einer der Adressatenkreise in die Irre geführt werde. Nachdem die Produkte freiverkäuflich waren und auch ohne jede Beratung online erworben werden konnten, könne auch nicht davon ausgegangen werden,

17) KG, Beschl. v. 22. 2. 2021 – 5 W 1024/20 –, GRUR-RS-2021, 8571.

18) OLG Düsseldorf, Beschl. v. 22. 3. 2021 – 15 U 3/21 –, GRUR-RS 2021, 19443.

19) OLG Hamm, Beschl. v. 2. 9. 2021 – 4 W 59/21 –, GRUR-RS 2021, 34494.

20) OLG Frankfurt a. M., Urt. v. 2. 12. 2021 – 6 U 121/20 –, PharmR 2022, 314 ff.

21) OLG Hamm, Urt. v. 21. 4. 2022 – 4 U 39/22 –, GRUR-RR 2022, 1083 ff.

dass eine Beratung und Aufklärung durch einen Arzt oder Apotheker stattfinde. Für die beworbene Wirkung konnte die Beklagte keine wissenschaftlich gesicherten Nachweise bringen. Entsprechend entschied der Senat auf Irreführung gem. §§ 3, 3a UWG, Art. 7 MDR.

*b) Zuwendungen*

Ebenfalls um Produkte der Wundversorgung ging es in der Entscheidung des OLG Hamm[22]. Die Herstellerin dieser Produkte warb gegenüber Apotheken und Arztpraxen als Dankeschön für den Einsatz im Rahmen der Corona Pandemie mit einem kostenlosen Online-Yoga-Kurs. Die Klägerin sah darin einen Verstoß gegen §§ 3, 3a UWG, 7 HWG. Der Senat teilte im Rahmen eines Hinweisbeschlusses mit, dass er in dieser Werbung keinen Produktbezug erkennen könne. Vielmehr handele es sich um eine Imagewerbung. Es fehle an jedem konkreten Bezug zwischen dem Online-Yogakurs und den von der Beklagten vertriebenen Produkte zur Wundversorgung. So bestünde schon kein Zusammenhang mit dem bereits erfolgten oder zukünftigen Bezug von Produkten der Beklagten. Ein solcher Produktbezug ließe sich auch nicht aus dem Satz „als Ihr Partner in der Wundversorgung“ herleiten. Mithin liege, so der Senat, kein Verstoß gegen § 7 Abs. 1 HWG vor.

Das OLG Nürnberg[23] hatte sich mit der Frage zu beschäftigen, ob die kostenlose Abgabe von FFP2-Schutzmasken einen Verstoß gegen die Coronavirus-Schutzmaskenverordnung (SchutzmV) darstellt. Ein Apotheker warb für die FFP2-Masken mit dem Slogan „6+1 FFP2-Masken gratis erhalten! 2,00 EUR Eigenanteil sparen!“. Der Senat sah darin einen Verstoß gegen § 6 SchutzmV. Dabei handele es sich um eine Marktverhaltensregel im Sinne des § 3a UWG. Die Vorschritt sei jedenfalls auch dazu bestimmt, dass Marktverhalten der Markteilnehmer zu regeln. Mangels expliziter Festlegung durch den Verordnungsgeber, sei der Normzweck durch Auslegung zu ermitteln, so der Senat. Der Referentenentwurf gebe den Hinweis, dass es das Ziel sei, allen anspruchsberechtigten Personen noch im Jahr 2020 die Möglichkeit zu geben, sich vor einer Infektion mit Hilfe von Schutzmasken zu schützen, weshalb der Anspruch auf eine bestimmte Anzahl beschränkt wurde. Hintergrund war, dass die FFP-2 Masken nur begrenzt verfügbar waren. Die Eigenbeteiligung solle zur verantwortungsvollen Inanspruchnahme beitragen. Es sollte mithin nicht nur das Kostenbewusstsein der Versicherten gestärkt werden. Vielmehr ginge es um eine verantwortungsvolle Inanspruchnahme der begrenzt verfügbaren Schutzmasken. Entsprechend diene die Vorschrift, so der Senat, damit einer Verhaltenssteuerung von Marktteilnehmern. Auf diese Weise, auch durch die Eigenbeteiligung, sollte verhindert werden, dass Berechtigte ohne Bedarf das Angebot nutzen. Die Eigenbeteiligung sollte auch tatsächlich durch die Apotheken eingenommen werden. All dies werde durch die kostenfreie Abgabe einer Maske unterlaufen, weshalb die Werbung einen Verstoß gegen eine Marktverhaltensregelung darstellt und mithin ein Verstoß gegen Lauterkeitsrecht, so das OLG. Das OLG Düsseldorf[24] kam zu einer anderen Auffassung als das OLG Nürnberg. Nach dem OLG Düsseldorf fehle der Regelung zur Eigenbeteiligung für Schutzmasken gem. Art. 6 S. 1 SchutzmV eine Schutzfunktion zu Gunsten anderer Marktteilnehmer. Daher handele es sich auch nicht um eine Marktverhaltensregel im Sinne von § 3a UWG. Konkret ging es um die Onlinewerbung einer Versandapotheke für die Ausgabe von FFP2-Masken. Die Apotheke warb damit, dass FFP2-Masken mit Berechtigungsschein kostenlos und versandkostenfrei erhalten werden konnten. Der Senat war der Auffassung, dass die Regelungen zur Zuzahlung gesetzlicher Versicherter bei Hilfsmitteln keine Marktverhaltensregelung gem. § 3a UWG sei. So lasse sich schon dem Wortlaut von § 6 SchutzmV keine Markverhaltensregelung unter Wettbewerbern entnehmen. Auch die „Entstehungsgeschichte der Vorschrift“ enthalte keinen Hinweis darauf. Der Hinweis in der Begründung des Referentenentwurfs, dass die Eigenbeteiligung zur verantwortungsvollen Inanspruchnahme beitragen soll, ändere daran nichts. Das Ziel einer flächendeckenden Versorgung vulnerabler Personengruppen mit den Schutzmasken werde nicht durch die in § 6 S. 1 SchutzmV normierte Eigenbeteiligung erreicht. Vielmehr regele dies die in §§ 2 bis 4 SchutzmV enthaltene mengenmäßige Begrenzung. Auch lasse sich, anders als die Vorinstanz dies annahm, der Verordnung nicht entnehmen, dass der Verordnungsgeber Wettbewerb zwischen Apotheken vermeiden wollte. Es gebe auch keine Anhaltspunkte dafür, dass die Eigenbeteiligung der Dispositionsbefugnis der Apotheke entzogen sei. Ferner diene zwar § 6 S. 1 SchutzmV auch der Verhaltenssteuerung von anspruchsberechtigten Personen, in dem diese zur verantwortungsvollen Inanspruchnahme angehalten werden sollen. Dies begründe aber kein Verbot der Apotheken, auf die Einziehung der Eigenbeteiligung zu verzichten. Der Senat sah in der Werbung auch keinen Verstoß gegen §§ 3, 3a UWG, 7 HWG. Er ließ dabei ausdrücklich offen, ob die FFP2-Maske Medizinprodukte sind. Die werbende Apotheke konnte sich nach dem Senat auf den Ausnahmetatbestand des § 7 Abs. 1 S. 2 Nr. 2 lit. a HWG berufen. Die Eigenbeteiligung stellt insoweit auch einen bestimmten Geldbetrag dar, nämlich 2 Euro je 6 Schutzmasken. Insgesamt erkannte der Senat in der entsprechenden Bewerbung damit keinen Verstoß der Apotheke.

In einem Streit zwischen zwei Herstellern von Produkten zur Behandlung von Inkontinenz hatte das OLG Köln[25] über die Zulässigkeit einer Testaktion zu urteilen. Die Beklagte bot auf den Produktverpackungen unter dem Slogan „Gratistest“ an, dass der Kaufpreis erstattet werde. Voraussetzung für die Kaufpreiserstattung war, dass ein Foto des Kassenbons und der Aktionspackung auf der Webseite der Beklagte hochgeladen wurde und die Kontaktdaten sowie die Bankverbindung angegeben wurde. Der Senat sah darin keinen Verstoß gegen § 7 Abs. 1 HWG. Zwar liege in der Kaufpreisrückerstattung eine unentgeltliche Vergünstigung und damit eine Zuwendung bzw. Werbegabe gem. § 7 Abs. 1 HWG. Der Beklagten stand jedoch der Ausnahmetatbestand § 7 Abs. 1 S. 1 Nr. 2 lit. a HWG zur Seite. Danach sind Zuwendungen zulässig, wenn es sich um einen bestimmten oder auf bestimmte Art zu berechnenden Geldbetrag handelt. Zuwendungsgegenstand sei in dem konkreten Fall nicht das Produkt, sondern vielmehr der zurückbezahlte Kaufpreis. In diesem Zusammenhang stellte der Senat auch darauf ab, dass nur ein geringfügiger Anteil (konkret 7 %) der Käufer den Kaufpreis überhaupt zurückverlangt hatten. Dies spreche dafür, dass der Verbraucher den Kaufpreis als Zuwendung ansehe, nicht das Produkt. Der Anwendbarkeit des Ausnahmetatbestands stehe auch nicht entgegen, dass der Kaufpreis vollständig erstattet werde. Die Regelung enthalte keine relative oder absolute Beschränkung in der Höhe des Geldbetrags. Nachdem bei einer vollständigen Kaufpreisrückzahlung auch nicht die Gefahr bestehe, dass durch eine Fehlvorstellung über den Wert der Zuwendung die Entscheidungsfreiheit der Abnehmer des Medizinprodukts beeinträchtigt werde, stehe dies auch nicht im Widerspruch zu der Geringwertigkeitsgrenze gem. § 7 Abs. 1 S. 1 Nr. 2 lit. a HWG, so das OLG.

22) OLG Hamm, Beschl. v. 13. 4. 2021 – 6 U 29/21 –, juris.
23) OLG Nürnberg, Beschl. v. 30. 9. 2021 – 3 U 2128/21 –, GRUR-RS 2021, 41051.
24) OLG Düsseldorf, Urt. v. 15. 4. 2021 – 15 Z 17/21 –, GRUR-RR 2021, 374.
25) OLG Köln, Urt. v. 17. 12. 2021 – 6 U 91/21 –, PharmR 2022, 310 ff.

Werden Medizinprodukte (hier: Wundversorgungsprodukte) mit einem „Gratis-Gutschein" für eine Fortbildung (hier: „Fachberater in Wundversorgung in der Apotheke") beworben, so liegt nach dem OLG Hamm[26] darin eine unlautere Zuwendung gem. § 7 Abs. 1 S. 2 HWG. Es handele sich um produktbezogene Werbung, und keine bloß allgemeine Firmenwerbung. Für die Einstufung sei auf das Gesamterscheinungsbild der Werbung abzustellen. Maßgeblich sei, ob die Darstellung des Unternehmens oder aber die Anpreisung bestimmter Produkte im Vordergrund stehe. Letzteres sei hier der Fall. Die Werbung diene erkennbar dem Absatz eines konkreten Produkts. Der Gratisgutschein werde nur bei Bestellung des Produkts ausgegeben. Hier liege auch eine abstrakte Gefahr einer unsachlichen Beeinflussung der Werbeadressaten. Die Fortbildung stelle einen persönlichen wirtschaftlichen Vorteil für die Apotheker dar. Der Senat wies insoweit auch daraufhin, dass die Werbende auf die Akkreditierungspunkte für die berufsrechtliche Fortbildung hinweise, die durch die Teilnahme erworben werden können. Es liege auch keine zulässige Werbegabe gem. § 7 Abs. 1 S. 1 Nr. 2 lit. a HWG vor. Unabhängig davon, dass im Rahmen der Werbung der Wert des Gutscheins in Euro angegeben werde, handele es sich dabei nicht um einen bestimmten oder auf bestimmte Art zu berechnenden Geldbetrag. Es handele sich lediglich um eine Sachzuwendung. Zudem liege darin auch keine handelsübliche Nebenleistung gem. § 7 Abs. 1 S. 1 Nr. 3. HWG oder eine Erteilung von Auskünften oder Ratschlägen gem. § 7 Abs. 1 S. 1 Nr. 4 HWG. Dies könnte allenfalls dann denkbar sein, wenn es sich bei der Fortbildungsveranstaltung um eine bloße Einweisung in das Produkt handele. Und auch die Ausnahmeregelung gem. § 7 Abs. 2 HWG sei nicht einschlägig, meinte der Senat. Die Übernahme von Teilnahmeentgelten für eine Veranstaltung sei ausdrücklich nicht erfasst.

## 4. Haftungsrecht

Im Rahmen eines Nichtzulassungsbeschwerdeverfahrens hatte sich der BGH[27] mit der Frage an die Anforderungen an den Vortrag zu medizinischen Fragen und Produkthaftungsfällen zu beschäftigen. In dem vom Senat zu entscheidenden Fall ging es um Ansprüche einer Patientin, die eine Durom-Oberflächenersatzprothese implantiert bekam. Sie machte Ansprüche gegen die Herstellerin wegen eines ausgeprägten Metallabriebs geltend, der zu einer bei der Klägerin entstandenen Metallbelastung durch Chrom und Kobalt geführt habe. Die Abriebmenge, so die Klägerin, sei bei dem Produkt der Beklagten deutlich über der zu erwartenden Menge gelegen. Die Klägerin hatte weder erstinstanzlich noch im Rahmen der Berufung mit ihrer Klage Erfolg. Nach Auffassung des Senats stellte das Berufungsgericht zu hohe Anforderungen an die Substantiierungspflicht der Klägerin. Darin liege ein Verstoß gegen rechtliches Gehör gem. Art. 103 Abs. 3 GG. Gerichte seien verpflichtet, tatsächliche und rechtliche Ausführungen der Beteiligten zur Kenntnis zu nehmen und in Erwägung zu ziehen, wobei die Anforderungen an die Substantiierung des Parteivortrags nicht überspannt werden dürfen. Der Senat führte unter Rückgriff auf die Kriterien im Arzthaftungsprozess aus, dass an die Substantiierungspflicht des Patienten nur maßvolle Anforderungen gestellt werden dürfen. Patienten haben regelmäßig keine genaue Kenntnis der medizinischen Vorgänge. Sie sind auch nicht verpflichtet, sich medizinisches Fachwissen anzueignen. Dies gelte entsprechend auch in Bezug auf Einwendungen gegen ein gerichtliches Gutachten. Parteien seien berechtigt, auch erstmal Einwendungen gegen ein Gutachten ohne sachverständige Hilfe vorzutragen. Im konkreten Fall habe die Klägerin unter Zugrundelegung dieser Anforderungen hinreichend substantiiert vorgetragen. Das Berufungsgericht wäre daher zur weiteren Sachaufklärung verpflichtet gewesen. Jedenfalls aber hätte es die Berufung nicht mit der Begründung zurückweisen dürfen, dass die Klägerin nicht substantiiert dem Gutachten entgegengetreten sei. Der Senat verwies den Fall zurück an das Berufungsgericht.

Das OLG Brandenburg[28] wies die Klage einer Patientin gegen die Herstellerin einer Hüfttotalendoprothese zurück. Der Senat sah die Ansprüche der Klägerin wegen eines Produktfehlers gem. § 3 ProdHaftG als nicht gegeben an. Der Senat konnte schon keinen sog. potentiellen Serienfehler erkennen. Zwar meinte der Senat, dass die Rechtsprechung zu Herzschrittmachern und implantierbaren kardioverten Defibrillatoren auch auf Fehlfunktionen von Hüftprothesen übertragen werden könne, auch wenn keine Todesgefahr drohe. Allerdings bestehe auch das Potential eines hohen Personenschadens im Hinblick auf implantierte Hüftprothesen. So würden Revisionsoperationen schwere körperliche Eingriffe darstellen, die die Übertragung der Rechtsprechung rechtfertigten. Im Ergebnis ließ der Senat dies jedoch offen. Die Ansprüche der Klägerin scheiterten schon daran, dass sie nicht aufzeigte, dass die Fehlfunktion tatsächlich bei einer signifikanten Anzahl von Produkten derselben Produktgruppe aufgetreten war. Die von der Klägerin vorgelegten Gutachten überzeugten den Senat nicht. Sie betrafen, so der Senat, andere Produktgruppen. Insbesondere seien die in Rede stehenden Keramikinlays unterschiedlicher Größen unterschiedlichen Produktgruppen zuzuordnen. Der Verkehr habe entsprechend auch unterschiedliche Sicherheitserwartungen an diese Produkte. Es liege auch weder ein Instruktionsfehler vor noch ein Fabrikationsfehler, so der Senat. Genauso wenig konnte das OLG einen Konstruktionsfehler feststellen. Aus der bloßen Tatsache, dass es zu einem Bruch eines Inlays kommen kann, lasse sich ein Konstruktionsfehler nicht begründen.

## 5. Recht der gesetzlichen Krankenversicherung

### *a) Anspruch des Versicherten auf Hilfsmittel nach §§ 33 ff. SGB V*

Die Hörgeräteversorgung der Versicherten mit Geräten oberhalb des Festbetrages ist regelmäßig mit wechselnden Akzenten Gegenstand der Rechtsprechung. In den letzten Jahren mussten sich mehrere Gerichte[29] mit der Frage befassen, welche Auswirkungen eine fünfprozentige Messdifferenz auf die Hörgeräteversorgung eines Versicherten hat. Der Freiburger Einsilbertest, der nach § 21 Hilfsmittel-Richtlinie für die beidohrige Hörgeräteversorgung vorgesehen ist, hat zuweilen ein um fünf Prozent besseres Sprachverstehen mit den Hörgeräten oberhalb des Festbetrages ergeben. Das LSG Baden-Württemberg[30] entschied erneut, dass ein fünfprozentiger Unterschied im Sprachverstehen der Messtoleranz zuzurechnen sei und insoweit kein messbarer Gebrauchsvorteil gegenüber einem zuzahlungsfreien Hörgerät gegeben sei, so dass es die Versorgung der Versicherten mit den von ihr begehrten Hörgeräten ablehnte.

Das Hessische LSG[31] bejahte zugunsten einer Versicherten die Versorgungsnotwendigkeit mit einer individuellen

26) OLG Hamm, Urt. v. 15. 2. 2022 – I-4 U 142/21 –, WRP 2022, 633 ff.
27) BGH, Beschl. v. 16. 2. 2021 – VI ZR 1104/20 –, PharmR 2021, 353 ff.
28) OLG Brandenburg, Urt. v. 16. 2. 2022 – 4 U 193/16 –, BeckRS 2022, 5801.
29) S. *Hobusch/Ochs*, MedR 2020, 911 ff., sub. 5. a.
30) LSG Bad.-Württ., Urt. v. 30. 11. 2021 – L 11 KR 3540/20 –, NZS 2022, 508.
31) Hess. LSG, Urt. v. 23. 9. 2021 – L 8 KR 477/20 –, juris.

Finger-Handprothese links aus Silikon als unmittelbaren Behinderungsausgleich. Bei der Versicherten besteht ein linksseitiger Teilhandverlust mit vollständig fehlendem Mittelfinger und ca. zur Hälfte vorhandenen Daumen, Zeige- und Ringfinger. Auf der Grundlage von eingeholten Sachverständigengutachten, der Anhörung der klagenden Versicherten und der Demonstration der Beweglichkeit der linken Hand durch die Versicherte kam das LSG zu dem Schluss, dass die verordnete und begehrte Teilhandprothese aus hochelastischem Silikon die Verbesserung der Greif-, Halte- und Führungsfunktion der linken Hand bewirke. Insbesondere könnten die Zangen-, Pinzetten-, Dreipunkt- und Schlüsselgriffe mittels Hilfestellungen durch die intakte rechte Hand zumindest näherungsweise simuliert werden. Ferner werde das (passive) Gegenhalten von Gegenständen, wie beispielsweise eines Telefons beim Eintippen von Daten mit der rechten Hand, verbessert. Ebenso sei davon auszugehen, dass die Versicherte beim Autofahren das Lenkrad mit der linken Hand gefahrlos halten könne, wenn sie die rechte Hand für z. B. die Betätigung von Armaturenbrettknöpfen einsetzen müsse.

In einem anderen Verfahren sah das Hessische LSG[32] zugunsten eines Versicherten die Versorgungsnotwendigkeit mit einem elektrischen Rollstuhlzuggerät (konkret: Handbike mit Elektromotor Typ „dynagil AP“) als gegeben an. Der klagende Versicherte verfügte über eine sehr geringe Greifkraft der Hände, so dass er die Greifringe seines Rollstuhls nicht ergreifen, sondern nur mit den Handballen schieben konnte. Dadurch waren ihm das Halten, Bremsen und das Fahren von Kippmanövern nahezu unmöglich, so dass er Bordsteinkanten und andere Hindernisse mit dem Rollstuhl nicht überwinden konnte. Das begehrte Rollstuhlzuggerät, das er eigenständig an den Rollstuhl ankoppeln kann, ermöglicht ihm dagegen sich den Nahbereich ohne fremde Hilfe zu erschließen, so dass das LSG die Betroffenheit eines im Rahmen des mittelbaren Behinderungsausgleichs relevanten allgemeinen Grundbedürfnisses sowie die Leistungspflicht der beklagten Krankenkasse bejahte.

Das LSG Berlin-Brandenburg[33] bejahte in einem einstweiligen Rechtsschutzverfahren den Anspruch einer klagenden Versicherten auf Versorgung mit einem restkraftunterstützenden elektrischen Greifreifenantrieb (konkret: „E-Motion M25“) zur Nutzung an dem vorhandenen manuellen Rollstuhl. Die Restarmkraft der Versicherten hatte sich infolge des progredienten Krankheitsverlaufs verringert, war tagesformabhängig und nicht seitengleich. Dadurch gelang es ihr beispielweise nicht, ohne fremde Hilfe Bordsteine zu überwinden. Durch den seitenungleichen Krafteinsatz bestand die Gefahr von Schlingerbewegungen. Da sich die Versicherte bei ihrer Versorgung mit dem begehrten Greifreifenantrieb den Nahbereich eigenständig ohne fremde Hilfe erschließen kann, sah das LSG die Hilfsmittelversorgung zulasten der Krankenkasse als notwendig an.

Nach einer Entscheidung des LSG Niedersachsen[34] steht eine zu 100 Prozent eingeschränkte Sehfähigkeit des Versicherten einer Versorgung mit einem Elektrorollstuhl nicht zwingend entgegen. In dem verhandelten Verfahren konnte sich das LSG durch Videoaufnahmen davon überzeugen, dass der Versicherte angesichts seiner Langstockerfahrung und der Orientierungs- und Mobilitätsschulung den Elektrorollstuhl sachgerecht bedienen und die bekannten Wege, inkl. der Überquerung einer Hauptstraße, bewältigen kann. Etwaige Restrisiken seien, so das LSG, der Eigenverantwortung des Versicherten zuzuordnen. Im Übrigen sah das LSG die Voraussetzungen für die Versorgung mit dem begehrten Elektrorollstuhl zur Erschließung des Nahbereichs als gegeben an. Der Versicherte konnte infolge seiner MS-Erkrankung nur noch wenige Schritte ohne Hilfe gehen. Einen Aktivrollstuhl konnte er aufgrund seiner eingeschränkten Armbeweglichkeit nur noch mit den Füßen und Trippelschritten bewegen, so dass er mit selbst kleine Hindernisse nicht mehr bewältigen konnte.

*b) Arzneimittelähnliche Medizinprodukte*

Versicherte haben zulasten ihrer Krankenkasse nur dann einen Anspruch auf (arzneimittelähnliche) Medizinprodukte, wenn sie gem. §§ 27–29 und Anlage V der Arzneimittelrichtlinie ausnahmsweise in den Leistungskatalog der gesetzlichen Krankenversicherung aufgenommen worden sind. Das Medizinprodukt Granudacyn Wundspüllösung gehört nicht dazu. In der Nichtaufnahme diese Medizinprodukts sah das LSG Baden-Württemberg[35] wie die Vorinstanz kein Systemversagen, so dass es die Berufung der Versicherten gegen den ablehnenden Gerichtsbescheid des SG zurückwies.

*c) Implantatversorgung*

Das BSG[36] hat erneut seine Rechtsprechung bestätigt, dass der in § 28 Abs. 2 S. 9 SGB V geregelte grundsätzliche Ausschluss der Implantatversorgung zulasten der Krankenkassen verfassungsgemäß ist. Ein Anspruch auf implantologische Leistungen besteht nur in den vom Gemeinsamen Bundesausschuss (G-BA) benannten Ausnahmeindikationen (vgl. dazu Abschnitt B. VII. der Richtlinie für eine ausreichende, zweckmäßige und wirtschaftliche vertragszahnärztliche Versorgung). Eine solche Ausnahmeindikation lag in dem vor dem BSG verhandelten Fall nicht vor. Der klagende Versicherte war zwar an Morbus Hodgkin (bösartiger Tumor des Lymphsystems) erkrankt. Jedoch konnte keine aufgrund der Strahlen- und Chemotherapie fortwirkende erhebliche Trockenheit im Mundbereich (Xerostomie) festgestellt werden. Ebenso wenig litt der Versicherte an größeren Kiefer- oder Gesichtsdefekten oder einer generalisierten genetischen Nichtanlage von Zähnen oder nicht willentlich beeinflussbaren muskulären Fehlfunktionen im Mund- und Gesichtsbereich (Spastiken).

*d) Hilfsmitteleinsatz im Rahmen einer neuen Untersuchungs- und Behandlungsmethode*

Gem. § 135 Abs. 1 SGB V darf eine neue Behandlungsmethode in der vertragsärztlichen Versorgung zu Lasten der Krankenkassen nur erbracht werden, wenn der G–BA die Methode anerkannt hat. Davon betroffen sind auch Medizinprodukte, die im Rahmen der Methode eingesetzt werden. Mangels Empfehlung des G-BA verneinte das LSG Nordrhein-Westfalen[37] den Anspruch eines minderjährigen Versicherten auf Versorgung mit orthokeratologischen Kontaktlinsen zur Behandlung einer Myopie. Die speziell angepassten formstabilen Kontaktlinsen werden nachts getragen und sollen bewirken, dass sich das Auge so verformt, dass die Myopie zurückgeht. In der fehlenden Empfehlung des G-BA sah das LSG auch kein Systemversagen, weil die Studienlage weitere Forschungen erfordere. Einen Ausnahmefall gem. § 2 Abs. 1a SGB V verneinte das LSG ebenfalls,

32) Hess. LSG, Urt. v. 25. 8. 2021 – L 1 KR 65/20 –, juris.
33) LSG Bln.-Brdbg., Beschl. v. 7. 4. 2022 – L 28 KR 8/22 B ER –, juris.
34) LSG Nds., Beschl. v. 4. 10. 2021 – L 16 KR 423/20 –, juris.
35) LSG Bad.-Württ., Urt. v. 25. 1. 2022 – L 11 KR 3215/21 –, juris.
36) BSG, Urt. v. 10. 3. 2022 – B 1 KR 2/21 R –, juris.
37) LSG NRW, Urt. v. 21. 6. 2021 – L 10 KR 303/20 –, juris; die klägerische Beschwerde gegen die Nichtzulassung der Revision verwarf das BSG als unzulässig, vgl. BSG, Beschl. v. 30. 11. 2021 – B 3 KR 25/21 –, juris.

weil keine lebensbedrohliche oder regelmäßig tödlich verlaufende oder mit einer solchen wertungsmäßig vergleichbaren Erkrankung vorlag.

Das Thüringer LSG[38] und das LSG Baden-Württemberg[39] kamen wie bereits andere LSG[40] zu dem Ergebnis, dass die Eigenanwendung des Bewegungstrainers Innowalk durch die Versicherten den Erfolg der Krankenbehandlung sichern solle. Das damit einhergehende intensivierte Funktionstraining sei jedoch Teil einer neuen Behandlungsmethode, für die die erforderliche positive Bewertung durch den G-BA fehle, so dass die Versorgung mit dem Bewegungstrainer von den Versicherten nicht beansprucht werden könne.

Gem. § 137e Abs. 7 SGB V kann der Hersteller eines Medizinprodukts, auf dessen Einsatz die technische Anwendung einer neuen Untersuchungs- oder Behandlungsmethode maßgeblich beruht, beim G-BA beantragen, dass dieser eine Richtlinie zur Erprobung der neuen Methode beschließt. Davon machte die Herstellerin eines Robotersystems in Form eines Exoskeletts zur Durchführung der sog. neuromuskulären Feedbacktherapie zur Behandlung von querschnittsgelähmten Patienten Gebrauch. Das neuronal gesteuerte Robotersystem verfügt über zwei bewegliche Beine, die die menschliche Bewegung imitieren können. Es zielt durch ein entsprechendes Training darauf ab, die Steh- und Gehfähigkeit der querschnittsgelähmten Patienten partiell wiederherzustellen. Der G-BA lehnte nach Einholung einer Stellungnahme beim Institut für Qualität und Wirtschaftlichkeit im Gesundheitswesen (IQWiG) den Erlass einer Erprobungsrichtlinie mangels Potenzial ab. Dem schloss sich das LSG Berlin-Brandenburg[41] an und wies die Klage der Herstellerin des Robotersystems als unbegründet ab. Das LSG führte aus, dass für die Rechtmäßigkeit der Potentialbewertung des G-BA spreche, dass die eine verfügbare, nicht vergleichende Studie mit sehr geringer Evidenz keine patientenrelevanten Vorteile im Sinne eines Potenzials erkennen lasse und das Patientenklientel der Behandlungsmethode nicht ausreichend konkretisiert sei. Der von der Herstellerin verwendete Begriff „austherapiert“ lasse nicht erkennen, welche Behandlungsmaßnahmen ohne Verbesserung des Gesundheitszustandes des Patienten abgeschlossen sein müssten, damit der Patient als austherapiert gelte.

Im Jahr 2017 beschloss der G-BA eine Richtlinie[42] zur Erprobung der Transkorneale Elektrostimulation zur Behandlung der Retinitis Pigmentosa (erblich bedingte Erkrankung der Netzhaut, die zu einer fortschreitenden Gesichtsfeldeinschränkung und letztlich zur Erblindung führt). Obwohl das Erprobungsverfahren noch nicht abgeschlossen war und somit die Anerkennung dieser neuen Methode durch den G-BA fehlte, bejahte das LSG Niedersachsen[43] (wie bereits das LSG Baden-Württemberg[44]) den Anspruch der klagenden Versicherten auf eine Versorgung mit dem bei der Behandlungsmethode zum Einsatz kommenden OkuStim®System (bestehend aus Gerät, Brille und Elektroden). Das LSG sah im Fall einer klagenden Versicherten angesichts der erheblichen Einschränkung des Sehvermögens und einer fehlenden Alternative einen Ausnahmefall gem. § 2 Abs. 1a SGB V als gegeben an.

### *e) Medizinprodukte im Kontext der Krankenhausvergütung*

Das LSG Baden-Württemberg[45] folgt der BSG-Rechtsprechung[46] zur Verknüpfung von Vergütung und Qualität der Krankenhausbehandlung und ist der Ansicht, dass ein Krankenhaus gegen das aus § 2 Abs. 1 S. 3 SGB V folgende Qualitätsgebot verstößt, wenn es ein Medizinprodukt (konkret: das atmungsunabhängige Hypoglossusstimulator System aura6000) außerhalb des vom Hersteller und von den Fachgesellschaften vorgesehen Anwendungsgebiets implantiert. Deshalb verneinte es den Anspruch des Krankenhauses auf Vergütung für eine Krankenhausbehandlung, die ausschließlich der Implantation des Hypoglossusstimulators diente. Das in Rede stehende Hypoglossusstimulator System ist bis zu einem die schlafbezogenen Atmungsstörungen widerspiegelnden Apnoe-Hypopnoe-Index (AHI) von 65/h indiziert. Die Patientin, der das Produkt implantiert worden war, hatte einen AHI von 83,4/h.

### *f) Hilfsmittelverzeichnis § 139 SGB V*

Das Asept Drainagekit, das an einen implantierten Katheter angeschlossen wird, um Flüssigkeit aus dem Brustraum abzuleiten, kann nach einer Entscheidung des LSG Berlin-Brandenburg[47] nicht für sich genommen in das Hilfsmittelverzeichnis (Produktgruppe geschlossene Absaugsysteme) eingetragen werden, weil es zusammen mit dem Implantat eine funktional untrennbare Einheit darstellt. Dem Drainagekit fehlt die Hilfsmitteleigenschaft, weil mit ihm allein der verfolgte Zweck, die Flüssigkeit abzuleiten, nicht erreicht werden kann. Dafür bedarf es immer dem Einsatz des Implantats.

**Open Access.** Dieser Artikel wird unter der Creative Commons Namensnennung 4.0 International Lizenz veröffentlicht, welche die Nutzung, Vervielfältigung, Bearbeitung, Verbreitung und Wiedergabe in jeglichem Medium und Format erlaubt, sofern Sie den/die ursprünglichen Autor(en) und die Quelle ordnungsgemäß nennen, einen Link zur Creative Commons Lizenz beifügen und angeben, ob Änderungen vorgenommen wurden.
Die in diesem Artikel enthaltenen Bilder und sonstiges Drittmaterial unterliegen ebenfalls der genannten Creative Commons Lizenz, sofern sich aus der Abbildungslegende nichts anderes ergibt. Sofern das betreffende Material nicht unter der genannten Creative Commons Lizenz steht und die betreffende Handlung nicht nach gesetzlichen Vorschriften erlaubt ist, ist für die oben aufgeführten Weiterverwendungen des Materials die Einwilligung des jeweiligen Rechteinhabers einzuholen.
Weitere Details zur Lizenz entnehmen Sie bitte der Lizenzinformation auf http://creativecommons.org/licenses/by/4.0/deed.de.

38) Thür. LSG, Urt. v. 23.12.2021 – L 6 KR 1126/18 –, juris; nachfolgend BSG B 3 KR 3/22 R.
39) LSG Bad.-Württ., Urt. v. 10.8.2021 – L 11 KR 4247/19 –, juris; nachfolgend BSG B 3 KR 8/21 R.
40) S. *Hobusch/Ochs*, MedR 2019, 865 ff., sub 5.b).
41) LSG Bln.-Brdbg., Urt. v. 20.5.2021 – L 14 KR 367/18 KL –, juris.
42) Erprobungs-Richtlinie TES-RP v. 20.7.2017, BAnz. AT 6.10.2017 B3, https://www.g-ba.de/richtlinien/99/.
43) LSG Nds., Urt. v. 13.12.2021 – L 4 KR 310/19 –, juris.
44) LSG Bad.-Württ., Urt. v. 18.2.2020 – L 11 KR 2478/19 –, juris; s. auch *Hobusch/Ochs*, MedR 2020, 911 ff., sub 5. b).
45) LSG Bad.-Württ., Urt. v. 22.2.2022 – L 11 KR 1308/20 –, juris.
46) Vgl. z.B. BSG, Urt. v. 1.7.2014 – B 1 KR 15/13 R –, BeckRS 2014, 72767; BSG, Urt. v. 25.3.2021 – B 1 KR 25/20 R –, BeckRS 2021, 19333.
47) LSG Bln.-Brdbg., Urt. v. 29.4.2021 – L 14 KR 48/18 –, BeckRS 2021, 33463; Revision anhängig: BSG B 3 KR 10/21 R.

Funding

Open access funding enabled and organized by Projekt DEAL.

